# Performance of Brazilian children on phonemic and semantic verbal fluency tasks

**DOI:** 10.1590/S1980-57642011DN05020004

**Published:** 2011

**Authors:** Helenice Charchat-Fichman, Rosinda Martins Oliveira, Andreza Morais da Silva

**Affiliations:** 1 Doutorado, Departamento de Psicologia, Pontifícia Universidade Católica do Rio de Janeiro, Rio de Janeiro RJ, Brazil; 2 Doutorado, Departamento de Psicometria, Instituto de Psicologia, Universidade ederal do Rio de Janeiro, Rio de Janeiro RJ, Brazil; 3 Psicóloga, Curso de Psicologia, Universidade Estácio de Sá, Rio de Janeiro RJ, Brazil

**Keywords:** child development, memory, neuropsychological tests, executive functions, language tests, cognition

## Abstract

The most used verbal fluency paradigms are semantic and letter fluency tasks. Studies suggest that these paradigms access semantic memory and executive function and are sensitive to frontal lobe disturbances. There are few studies in Brazilian samples on these paradigms.

**Objective:**

The present study investigated performance, and the effects of age, on verbal fluency tasks in Brazilian children. The results were compared with those of other studies, and the consistency of the scoring criteria data is presented.

**Methods:**

A sample of 119 children (7 to 10 years old) was submitted to the three phonemic fluency (F, A, M) tasks and three semantic fluency (animals, clothes, fruits) tasks. The results of thirty subjects were scored by two independent examiners.

**Results:**

A significant positive correlation was found between the scores calculated by the two independent examiners. Significant positive correlations were found between performance on the semantic fluency task and the phonemic fluency task. The effect of age was significant for both tasks, and a significant difference was found between the 7- and 9-year-old subjects and between the 7- and 10-year-old subjects. The 8-year-old group did not differ to any of the other age groups.

**Conclusion:**

The pattern of results was similar to that observed in previous Brazilian and international studies.

## Abstract (pt)

Os paradigmas de fluência verbal mais usados são tarefas de fluência verbal fonêmica e por categoria semântica. Estudos indicam que estes paradigmas acessam a memória semântica e as funções executivas. Existem poucos estudos com amostras brasileiras sobre estes paradigmas.

**Objetivo:**

Investigar o desempenho e efeito de idade de crianças brasileiras em paradigmas de fluência verbal, comparar os resultados com estudos estrangeiros e avaliar a consistência dos critérios de pontuação entre examinadores.

**Métodos:**

Uma amostra de 119 crianças, entre 7 e 10 anos de idade, foi submetida a três tarefas de fluência por letra (F, A, M) e três de fluência semântica (animais, roupas, frutas). Trinta protocolos foram pontuados por dois examinadores independentes.

**Resultados:**

Observou-se correlação positiva e significativa entre os escores calculados pelos dois examinadores independentes. Também verificou-se uma correlação positiva e significativa entre as tarefas de fluência semântica e entre as tarefas de fluência por letra. O efeito de idade foi significante para os dois tipos de tarefa e observou-se diferença significativa entre o desempenho dos grupos de 7 e 9 anos, assim como entre 7 e 10 anos de idade. O desempenho do grupo de 8 anos não foi diferente dos outros grupos.

**Conclusão:**

O padrão de resultados observado é comparável a estudos estrangeiros.

## Introduction

Fluency of speech is typically measured by the quantity of words produced under restricted search conditions within a limited time. Laine and Niemi^1^ defined two kinds of conceptual clustering that have similar features: phonological clusters, which share the same initial sound group of letters, and semantic clusters associated by meaning. Two types of fluency tasks are commonly used: semantic and phonemic fluency. In semantic fluency tasks, the subject must produce, within a limited time interval, as many words as possible belonging to a given semantic category. In phonemic fluency tasks, the words to be produced must begin with a given letter.^2^

The first version of the verbal fluency test was Controlled Oral Word Association proposed by Benton.^3^ The most commonly used form is FAS, a three-word-naming trial included in the Neurosensory Center Comprehensive Examination of Aphasia^2^ and the Iowa Screening Battery for Mental Decline.^4^ The selection of the FAS letters depends on vocabulary frequencies of English words beginning with these letters. Other forms are employed in Multilingual Aphasia Examinations, such as CFL, SNF, or PRW.^3^

The most common semantic fluency test uses the “animals” category. This task is included in the Boston Aphasia Examination, Stanford-Binet Intelligence Scale, and Clinical Evaluation of Language Fundamentals-3. Other categories used are “things in the kitchen,” “supermarket,” and “alternations of colors and birds”.^2^

Akin to studies in adult subjects, studies in children have reported that the number of words produced is greater in semantic than in phonemic fluency tasks.^5-8^ These findings are explained in terms of a greater dependency of phonemic fluency on executive functions, such as cognitive flexibility and shifting, compared with semantic tasks. Semantic task output appears to be more related to lexical-semantic networks comprised in semantic memory and comparatively less dependent on executive function.^9,10^

The differences in developmental courses of phonemic and semantic fluencies also corroborate this understanding about cognitive processes and structures underlying performance on these tasks. Performance on both tasks improves throughout childhood and adolescence. However, although phonemic fluency continues to change in later childhood and adolescence, changes in semantic fluency are more pronounced in early childhood, becoming relatively stable after 10-12 years of age.^5,6^ The improvement in semantic fluency performance may result from lexical-semantic memory development. In contrast, the comparatively delayed age difference in performance on phonemic tasks may be explained in terms of the development of strategic search abilities, such as cognitive flexibility and shifting,^5,6^ which depend on the development of the central executive and maturation of the frontal lobes, which peak at ages of between 10 and 12 years.^11-13^

Verbal fluency tasks are used in clinical and research settings to assess processes of strategic search and retrieval from semantic memory, in addition to the content of this system. Impairment in verbal fluency task performance is associated with frontal lobe dysfunction.^9^ These tasks are related to frontal and temporal lobe activation^14,15^ and are sensitive to frontal lobe dysfunction during childhood, including attention-deficit/hyperactivity disorder,^16,17^ autism,^18^ Tourette’s syndrome,^19^ and Turner’s syndrome.^20^

The verbal fluency paradigm has been used for years in Brazil, primarily in studies with adult samples that have focused on aging and neurological and psychiatric diseases, such as dementia and multiple sclerosis.^21,25^ A few studies have also been conducted in children.^26,29^ In two such studies, fluency tasks were used to assess the level of functioning of the frontal lobes in neurologically affected children.^27,28^ Two other studies investigated the performance of normal children on semantic fluency tasks during childhood.^26,29^

The present study assessed the performance of 119 Brazilian children on three phonemic fluency tasks and three semantic fluency tasks, analyzed the effects of age on performance, and compared these results with previous national and international studies. The consistency of the scoring criteria was also examined.

## Methods

### Subjects

One hundred nineteen subjects participated in the study, including 59 boys and 60 girls, ranging in age from 7 to 10 years. The Chi-square test showed no significant difference between age groups in terms of gender frequencies (p>0.05). Results on the Chi-square test comparing education grade frequencies at different ages were significant (p<0.05) (Table 1). The subjects assessed were students from private schools in Rio de Janeiro. The subjects belonged to socioeconomic classes C and D, estimated from their parents’ professions and reported income. All children in the age range of interest were included as subjects, with the exception of those with diagnosed neurological or neuropsychiatric disturbances determined by a questionnaire completed by their parents. For all subjects, written informed consent was obtained from their parents, and the study was approved by the local institutional ethics committee.

**Table 1:** Subject characteristics for each age group.

	7 years	8 years	9 years	10 years	p-value
N	37	28	34	20	
Gender					
Female	20	10	19	11	>0.05^a^
Male	17	18	15	9	
Education					
1^st^ grade	4	0	0	0	
2^nd^ grade	23	13	0	0	
3^rd^ grade	1	10	10	1	<0.05^b^
4^th^ grade	0	0	22	5	
5^th^ grade	0	0	2	14	

a χ^2^ test comparing gender frequencies for different ages.

b χ^2^ test comparing education grade frequencies for different ages.

### Procedures

The subjects were tested individually in vacant rooms in the schools. The material used was paper, a pencil, and an audio tape recorder to record the subject’s responses. Each participant performed three phonemic fluency tasks and three semantic fluency tasks. For phonemic fluency, participants were given the letters F, A, and M and were asked to generate as many words as possible beginning with these letters within a 60 s interval, excluding proper names and repetitions of the same words with different endings. For semantic fluency, participants were given the categories animals, fruits, and clothes. For each of these three semantic categories, the subject was instructed to name as many different members of the category as possible within a 60 s interval (Strauss et al., 2006). The presentation order of the tasks was the same for all subjects: phonemic fluency (F, A, M) followed by semantic fluency (animals, fruits, and clothes).

The number of correct words produced in each task was tallied for each of the six tasks. Errors were excluded from the total word count according to the set of criteria shown in Boxes 1 and 2. The number of errors was also counted for each task. The responses of a sample of 37 subjects were evaluated by two independent examiners to determine the internal consistency of the scoring criteria shown in Boxes 1 and 2.

**Box 1:** Criteria for counting the number of correct words in phonemic fluency tasks.

Emissions counted as errors:	
•	Words not beginning with the appropriate letter.
•	Repetition of a correct word produced previously in the same task by the subject.
•	Proper nouns (people's names, countries, cities, car makes, etc.).
•	Words that cannot be found in the Portuguese dictionary *Novo Dicionário Aurélio da Língua Portuguesa* (Ferreira, 2004).
•	A word that is no more than a variation of a correct word previously produced by the subject in terms of number (singular or plural), size (augmentative, diminutive, superlative), gender (masculine, feminine, with the change of the ending of the word), and verb conjugation (differing only in person or tense).
•	A verb with a root that is the same as the name or adjective produced previously and accepted as correct and vice-versa (e.g., *fim* [the end] and *finalizar *[to end], *amor *[love] and *amar *[to love], *mendigo *[beggar] and *mendigar *[to beg], *admirar *[to admire] and *admirável *[admirable]).
Emissions not counted as errors:	
•	Names with the same root but with different meanings (e.g., *faca* [knife] and *faqueiro *[cutlery], *filho *[son] and *filhote *[cub], *faminto *[hungry] and *fome *[hunger], *fazenda *[farm] and *fazendeiro *[farmer]).
•	Many words have more than one meaning (e.g., *tomada* [plug], *pé *[foot], and *ralo *[drain]). If the subject repeats a word but indicates spontaneously or after inquiring that a second meaning was intended, then this is not considered a repetition error (e.g., the subject says *ralo *twice but explains that the first emission refers to *drain* and the second refers to *diluted*; these two emissions of *ralo *are counted).
•	Slang words are acceptable if they are commonly used.

**Box 2:** Criteria for counting the number of correct words in semantic fluency tasks.

1.	General criteria for all semantic tasks	
	Emissions counted as correct:	
	•	Words belonging to the semantic class designated in the task.
	•	Words that can be found in the Portuguese dictionary *Novo Dicionário Aurélio da Língua Portuguesa*.^30^
	•	If the subject produces the name of a general category (e.g., bird, primate, mammal, plant) and also says specific items belonging to that category, then only the specific items are counted and not the general category (e.g., *bird* [general category], pigeon [specific item], parrot [specific items]).
	Emissions counted as errors:	
	•	A repetition of a correct word produced previously in the same task by the subject.
2.	Specific criteria for clothing	
	Emissions counted as correct:	
	•	Clothes and accessories used to cover or adorn the body.
	•	Some examples of correct emissions that may cause doubt include *relógio* (watch), *jóia *(jewelry), *fita *(hair ribbon), headdresses such as *turbante *(turban), *lenço* (headscarf), *tiara *(tiara), *óculos *(glasses), and *peruca *(wig).
	•	*Casaco *(coat), *blazer *(blazer), *agasalho, sueter, jaqueta *(sports jacket), *sobretudo *(overcoat), and *paletó *(jacket) are considered different words.
	Emissions counted as errors:	
	•	Errors as specified in item 1 above.
	•	Some examples of errors that may cause doubt include *botão* (button), *cardaço *(shoe lace), *carteira *(wallet), *bolsa *(bag), *guarda-chuva *(umbrella), and *bengala *(cane).
3.	Specific criteria for animals	
	Emissions counted as correct:	
	•	Names of different dinosaurs or the word *dinossauro* (dinosaur).
	Emissions counted as errors:	
	•	Mythical animals, such as *unicórnio* (unicorn).

### Statistical analysis

Initially, the internal consistency of the complete set of results was tested using Pearson’s correlations between tasks. The effect of type of fluency (i.e., phonemic and semantic) was tested using one-way analysis of variance (ANOVA) for the number of correct words. Differences among the three tasks for each type of fluency were analyzed by t-tests. The effect of age was determined using ANOVA followed by the Least Significant Difference (LSD) *post hoc* test and Pearson’s correlation between age (in months) and the different scores. Finally, the internal consistency of the scoring criteria was tested using Pearson’s correlation between the scores assigned by the two independent examiners.

## Results

To investigate the consistency of the verbal fluency scoring criteria, Pearson’s correlation analysis was performed between the scores found by the two independent examiners for 37 protocols. A significant positive correlation was found between the examiners for the number of correct words for each task: “F” (r=0.963, p<0.01), “A” (r=0.971, p<0.01), “M” (r=0.953, p<0.05), animals (r=0.883, p<0.01), fruits (r=0.898, p<0.01), and clothes (r=0.903, p<0.01).

The multivariate ANOVA showed a significant effect of condition on the verbal fluency tasks (F_4_=18.88, p<0.05). The number of correct words produced was greater in the semantic fluency tasks than in the letter fluency tasks. A significant correlation was found between the letter fluency tasks (0.50<0.63, p<0.05) and between the semantic fluency tasks (0.33<0.63, p<0.05) and between the semantic fluency tasks (0.33<0.39, p<0.05). A significant positive correlation was observed between the “F” (r=0.30, 0.30, 0.28, p<0.05), “A” (r=0.24, 0.49, 0.32, p<0.05), and “M” (r=0.34, 0.37, 0.45, p<0.05) letter tasks and the animals, fruits, and clothes semantic fluency tasks, respectively. The total number of errors in semantic fluency correlated with total errors in letter fluency (r=0.861, p<0.05).

Table 2 shows the results for the three letter fluency tasks and the three semantic tasks (means and standard deviations). The number of correct words for animal semantic verbal fluency was higher than for fruits (t=8.1, p<0.05) and clothes (t=7.6, p<0.05). No difference was found between the letter fluency tasks.

**Table 2:** Mean and standard deviation of verbal fluency tasks (number of correct words).

Verbal fluency tasks	Mean	SD
"F"	6.47	2.89
"A"	6.11	2.63
"M"	6.34	2.94
Animals	11.07	3.49
Fruits	8.45	3.32
Clothes	8.49	2.54

The effects of age (7, 8, 9, and 10 years) and condition (letter fluency: “F,” “A,” and “M”; semantic fluency: “animals,” “fruits,” and “clothes”) were tested using ANOVAs for each of the scores studied. The ANOVA showed a significant effect of age (F=2.23, p<0.01). An effect of age was also found on the number of correct words on all letter and semantic tasks (p<0.05), but no effect of age was found on the number of errors (p > 0.05). The interaction between age (in years) and type of fluency task (letter fluency and semantic fluency) was not significant for either of the two scores. Means, standard deviations, F values, and p values are shown in Table 3.

**Table 3:** Effects of age on verbal fluency tasks.

Verbal fluency tasks	Age (years)					p value
	7 Mean (SD)	8 Mean (SD)	9 Mean (SD)	10 Mean (SD)	F	
"F"	5.08 (2.13)	6.31 (3.38)	7.72 (2.62)	7.35 (2.73)	6.40	p<0.01
"A"	4.78 (1.94)	5.06 (2.75)	7.12 (2.73)	7.65 (2.00)	8.87	p<0.01
"M"	4.76 (2.34)	6.86 (3.47)	7.24 (2.65)	7.10 (2.59)	6.03	p<0.01

The LSD *post hoc* test showed that 7-year-old subjects produced fewer correct responses than 9-year-olds (p<0.05) and 10-year-olds (p<0.05) on all of the letter and semantic fluency tasks. The 8-year-old group did not perform differently compared with the other age groups (p>0.05) on any of the verbal fluency tasks.

Another way of studying the age factor was to test the correlation between verbal fluency performance and age in months rather than years. The transformation of the age categories (discrete variable) into months (i.e., a more continuous variable) has the advantage of allowing the study of changes over shorter time intervals.

A significant positive Pearson correlation was found between age (in months) and the number of correct words: “F” (r=0.34, p<0.05), “A” (r=0.31, p<0.05), “M” (r=0.26, p<0.05), animals (r=0.37, p<0.05), fruits (r=0.32, p<0.05), and clothes (r=0.311, p<0.05). A significant correlation was also found between age (in months) and the sum of the number of correct words of the three letter verbal fluency tasks (r=0.36, p<0.05) and the sum of the three semantic verbal fluency tasks (r=0.44, p<0.05). The number of errors did not correlate with age (in months) on either the semantic (p>0.05) or letter verbal fluency tasks (p>0.05). The multivariate ANOVA showed no effect of gender and no interaction between gender and type of fluency.

## Discussion

The scoring criteria for verbal fluency tasks have a high degree of subjectivity,^2^ but the examination of the scoring consistency between the examiners in the present study showed that the criteria developed for the present study were reliable. The results showed internal consistency since significant correlations were found among all fluency tasks. The number of errors was very small, and no significant effects were observed for this score. This is a common finding in studies using subjects from this age range.^5,6^

The pattern of the present results is similar to those reported by previous studies. The number of correct words produced was greater in semantic than in letter tasks.^5,7^ This pattern may be explained in terms of the greater requirements of executive function for letters compared with semantic fluency. In semantic fluency, the subject recruits a semantic network in which internal connections help guide the word search. In letter fluency, the subject must organize the search by themselves and sample from different categories, an operation requiring more strategic retrieval.

A significant difference was found among the three semantic tasks, whereas no differences were found among the three letter tasks in terms of number of correct words. Similarly, other studies have shown no differences among various letter sets, despite selecting letters based on the analysis of letter difficulty determined by the number of words in English beginning with that particular letter.^2^ However, differences between semantic categories have been found and may be explained in terms of category familiarity.^7^

No effect of gender was observed in the present study, which is consistent with other Brazilian^26,29^ and foreign studies^5,7,8^ in children. Data from adults are more variable in terms of possible gender effects,^2^ but in studies with children, the effect of gender is sometimes observed but only for clustering scores.^5,26^

An effect of age was found for all of the semantic and letter fluency tasks. Seven-year-old subjects produced fewer words than the 9- and 10-year-old children, while no difference was observed between the 8-year-old subjects and the other age groups. Additionally, significant correlations were found between age (in months) and the number of correct words for each of the six conditions. The effect of age on fluency performance is consistent with other studies, including data from studies involving Brazilian children. The significant differences in performance between the 7- and 10-year-old subjects, but not between the 7- and 8-year-old subjects or between the 9- and 10-year-old subjects, are similar to those found by Malloy-Diniz and colleagues^29^ who also employed a Brazilian sample. These results indicate that 8 to 9 years of age may be a transition phase in the development of executive function in childhood. The investigation of the executive function is a useful tool for cognitive rehabilitation and early diagnosis of neuropsychiatric disturbances of infancy.

The Brazilian sample studied by Malloy-Diniz and colleagues^29^ produced more animal names than the sample investigated in the present study. One possible reason for this discrepancy may be the specific and restrictive criteria used to count correct responses and errors employed in the present study (Box 2). Another explanation may be the demographic or regional characteristics of the sample (i.e., one collected in Rio de Janeiro and the other collected in Belo Horizonte, Minas Gerais). Finally, another possible factor may be the inclusion criteria for the sample selection. Malloy-Diniz and colleagues^29^ included only children with a percentile of above 25 on Raven’s progressive matrices, whereas the present study included all children, with the exception of those presenting with neurological or psychiatric disturbances.

The interaction between age (in years) and type of fluency was not significant, which is consistent with other studies that used a broader span of ages. One might expect to find variations in the effect of age on type of fluency in older children because semantic fluency is more pronounced in early childhood, becoming stable at 10 to 12 years of age, whereas letter fluency continues to change in later childhood and adolescence^5,6^ when the development of the central executive and the maturation of the frontal lobes peak.^11,13^
